# You Reap What You Sow: Customer Courtesy and Employees’ Prosocial Service Behavior

**DOI:** 10.3390/bs14090736

**Published:** 2024-08-24

**Authors:** Cuicui Pan, Hyung-Min Choi

**Affiliations:** 1 Department of Hotel and Tourism Management, The Graduate School, Youngsan University, Haeundae Campus, Busan 48015, Republic of Korea; choipan@office.ysu.ac.kr; 2 Department of Foodservice Management, College of Hotel and Tourism, Youngsan University, Haeundae Campus, Busan 48015, Republic of Korea

**Keywords:** customer courtesy, prosocial service behavior, organization-based self-esteem, focus of attention at work, hospitality industry

## Abstract

Smooth and effective interactions between customers and customer-contact employees are important for building seamless service delivery. The present study examined the influence of customer courtesy on customer-contact employees’ prosocial service behavior. Additionally, the mediating role of organization-based self-esteem and the moderating role of focus of attention at work are investigated. Data were collected from 401 customer-contact employees from the South Korean hospitality industry. The results demonstrated that customer courtesy positively influenced prosocial service behavior, and organization-based self-esteem mediated this relationship. Furthermore, employees’ focus of attention at work moderated the relationship between customer courtesy and organization-based self-esteem, such that the relationship was stronger for employees with a high focus of attention. Similarly, the focus of attention also moderated the relationship between organization-based self-esteem and prosocial service behavior. The findings have important theoretical and practical implications by demonstrating the role of external resources to promote prosocial service behavior.

## 1. Introduction

The focus of the global economy has increasingly moved from manufacturing to services. Excellent service quality is now one of the key factors impacting an organization’s performance and success [1]. Accordingly, “*service quality or service excellence as a strategic requirement or, at the very least, a strategic opportunity*” is seen by virtually all firms [2]. High-quality service interactions between customer-contact staff and their customers are one of the most popular strategies to seek exceptional service quality [3,4]. Previous research has revealed that service quality can be improved when customer-contact employees go the extra mile for their clients [5]. An employee’s prosocial behavior is their willingness to help others (coworkers and organization) when they interact with them in service encounters [6]. Investing in the development of prosocial service behaviors (PSSBs) among hospitality staff is crucial. Such behaviors demonstrably enhance customer perceptions of “*service quality*” and “*satisfaction*” (e.g., [7,8]), which are essential for the success of a hospitality business (e.g., [9,10]). Consequently, PSSBs have significant ramifications for the hotel industry.

Although the hospitality industry places a high priority on PSSBs, further study is required to identify the drivers that influence customer-contact employees’ PSSBs [10]. While several studies investigated the factors that negatively affect employees’ PSSBs [11,12], only a handful of studies examined the factors positively influencing PSSBs in the hospitality industry (e.g., [5,10,13,14]). At the same time, in the wake of a shift in the perspective of scholars toward seeing customers as active participants in shaping service experience, scholars are keen to examine how customers’ attitude and behavior toward customer-contact employees influences their PSSB, along with the factors that mediate and moderate this relationship [15]. This study fills the current gap by investigating “how” and “whom” customer courtesy, that is, a customer’s favorable behavior and positive emotional display directed toward a customer-contact employee, influence employees’ PSSBs.

Since customer courtesy was not recognized as a separate construct until recently [15], empirical research in this area is still in its infancy. To understand the mechanism through which customer courtesy impacts PSSB, organization-based self-esteem (OBSE) as a mediator and the moderating role of focus of attention at work (FAW) are investigated. OSBE represents the self-esteem that is specific to organizational contexts and reflects “*the self-perceived value that individuals have of themselves as organization members acting within an organizational context…employees with OBSE perceive themselves as important, meaningful, effectual, and worthwhile within their employing organizations*” [16]. Gardner et al. defined an employee’s FAW as how employees think about each of the many targets they must attend to while at work [17]. In other words, an employee’s mental impressions of objects, events, and phenomena while physically present at work are what and how strongly they are felt.

The study contributes to the hospitality literature in several ways. Customers may be the most frequent sources of feedback for customer-contact staff given the amount of time they spend with them; thus, it is crucial to understand the influence of these customer interactions. The study responds to Subramony et al.’s and Yoon et al.’s calls for further research on how customers’ positive responses/behavior toward employees might affect employees’ attitudes and behaviors [15,18]. The study further broadens the literature by understanding the predictors of PSSB beyond organizational boundaries. Furthermore, the findings contribute to the limited literature by exploring a unique context characterized by South Korea’s collectivist culture and hierarchical social structure. These cultural elements significantly influence customer–employee interactions, where respect, politeness, and non-confrontational communication are highly valued. Additionally, the rapid expansion of the hospitality industry in South Korea, driven by both domestic and international tourism, presents distinct challenges and opportunities that differ from those in Western contexts.

## 2. Hypotheses Development

### 2.1. Customer Courtesy and OBSE

Yoon et al. introduced the construct “customer courtesy” and defined it as “customers being cooperative with service employees on a matter over which employees have some responsibility, being polite and warm to employees, and displaying positive mannerisms towards employees [15]”. A customer’s courtesy is reflected in the way they treat employees. Customer courtesy transcends mere positive verbal expressions. It manifests rather as a product of a transient, positive interaction between employees and customers. In such interactions, customers may exhibit favorable emotions toward the employee, but these emotions are not necessarily rooted in a long-term relationship [19,20,21].

Pierce et al. proposed the construct OBSE, and they defined it as “the degree to which an individual believes himself/herself to be capable, significant, and worthy as an organizational member [16]”. Employees with high OBSE often cultivate a strong sense of self-worth within the organization. This sense of self-worth can lead them to engage in self-evaluation of their own contributions. OBSE is the process through which individuals evaluate their performance in light of organizational needs [22]. According to earlier studies on the predictors of self-esteem [23,24], the development of OBSE is largely determined by how important and significant others at work (for example, supervisors or coworkers) tell one about one’s competence and how one feels competent because of one’s own experiences [22,25].

We presume customer courtesy is positively related to OBSE for the following reasons: Customers have a significant impact on a hospitality firm’s businesses and their staff, as they are considered the most significant stakeholders in the hospitality industry [26,27]. Customers responding politely to employees’ conduct gives employees a signal that their behaviors have produced the desired response and serves as recognition of their abilities and efforts [28,29]. Customers’ courteous behavior can be considered a sign of reciprocation based on Gouldner’s norm of reciprocity [30], and as such, it provides evidence that the employees were displaying courteous behavior themselves and were upholding the fundamental expectation of a service employee [15]. When customers treat employees with politeness and respect, employees see themselves as competent, likable, and respectable [31,32], thus positively influencing their OBSE. Similarly, Dong et al. and Martinaityte et al. noted that the customers’ behavior during service interactions conveys information on how well the employees are performing their duties [33,34]. The positive response from customers indicates that an employee has performed his/her job successfully and fosters a sense of achievement. Service interactions are considered goal attainment situations for customer-contact employees, and customers’ civil behavior may be viewed as an instance of task success [35,36]. Also, from a social psychology perspective, Ilgen et al. noted that positive responses from significant others are “*seen as a necessary component of task environments by those who emphasize the importance of higher order needs for self-esteem and self-actualization* [37]”. The significance of antecedents in OBSE has been echoed in the literature exploring similar domains, suggesting a broad consensus among scholars regarding their impact (e.g., [38]). Furthermore, the intersection of social psychology with organizational behavior has illuminated how positive feedback from significant others can serve as a crucial foundation for employees’ self-esteem within the workplace. This understanding draws upon foundational theories and pioneering research [39,40,41], highlighting the importance of social interactions and perceptions in shaping self-esteem. Therefore, it is anticipated that customer courtesy will enhance employees’ sense of self-efficacy and increase their OBSE. Accordingly, we hypothesize the following:

**H1.**  *Customer courtesy will positively impact employees’ OBSE*.

### 2.2. OBSE and PSSB

In general, prosocial behaviors are positive behaviors displayed by service employees toward customers and coworkers [6,42]. They are characterized by discretionary actions that exceed the formal job requirements of employees. These behaviors aim to enhance the customer experience and can include offering unexpected “extras” that “delight” the customers, treating them with exceptional courtesy, or going the “extra mile” to fulfill their needs [43,44]. Beyond their immediate positive impact on customer interactions, cultivating prosocial behaviors among employees offers substantial benefits for businesses, especially in service-oriented industries like hospitality. Prosocial behaviors, such as going above and beyond for customers, are directly linked to enhanced customer satisfaction, which, in turn, fosters customer loyalty and repeat business [6,45]. Satisfied customers are more likely to share positive experiences, leading to valuable word-of-mouth marketing and a stronger brand reputation [46,47,48]. Additionally, when employees regularly engage in prosocial behaviors, it contributes to a more positive and collaborative workplace culture, which can reduce employee turnover and improve morale [49,50]. A workplace where employees feel valued and motivated to contribute positively often sees higher levels of employee engagement and productivity [51,52,53,54]. Ultimately, these factors combine to improve overall business performance, making the cultivation of prosocial behaviors not just a beneficial practice but a strategic imperative for long-term success in the competitive hospitality industry. When analyzing the possible outcomes of OBSE, Pierce et al. pointed out that “*cognitive consistency theory suggests that people are driven to obtain results that are consistent with their self-concept* [55]” [16]. They also claimed that those with a high OBSE, or those who believe they are capable, important, and relevant to the organization, will be innately motivated to act in ways that support that perception and experience fulfillment in carrying out tasks that the organization values. Scholars have proposed a positive link between OBSE and several job-related outcomes [56,57,58]. Similarly, Kim and Beehr demonstrated that employees’ OBSE was positively related to citizenship behaviors toward co-workers and organizations [59]. Specifically, Park and Kim demonstrated the positive association between OBSE and employees’ service performance in the hospitality context [27]. In a similar vein, we propose the following:

**H2.**  *OBSE has a positive influence on PSSB*.

### 2.3. Customer Courtesy and PSSB

Frontline employees’ behaviors are not only influenced by organizational practices but also by customers’ behavior [60,61]. For instance, they considered customers “partial employees” because of the crucial role they play in the service process [62]. The influence of customers’ behavior on service employees’ attitudes is reinforced when customers are viewed as members of the service team. Several studies have suggested that pleasant customer behavior can co-create a pleasant service experience [63,64]. Therefore, we believe that positive customer behavior, such as customer courtesy during service encounters, can positively influence employees’ psychological responses (e.g., PSSB). Building on the concept of social exchange [65], the principle of reciprocity helps explain why customer-contact employees who experience positive treatment from customers feel compelled to reciprocate in kind. Based on Lawler’s “*affect theory of social exchange*”, the service-profit chain model illustrates how positive social interactions create positive feelings and may inspire employees to surpass expectations [66]. In light of this, when customers treat employees with courtesy, it suggests the employees have the psychological resources to take extra care of the customers. When individuals engage in a social exchange relationship, experiencing positive emotions is perceived to be rewarding [66,67]. It is particularly critical to recognize that social exchanges have profound effects when individuals believe they are jointly accountable for the success or failure of their exchange. Dadfar et al. and Gazzoli et al. noted that a similar dynamic occurs with hotel service delivery, where customer-contact employees and clients share equal responsibility in creating the overall service experience [68,69]. Consequently, when customer-contact employees interact with courteous customers and have a satisfying service experience, they feel obliged to compensate for any good deeds they receive. This obligation may be intensified in social exchange relationships when the recipient of good actions is subservient to the donor (such as service staff) [70]. Drawing from the above discussions, we propose the following:

**H3.**  *Customer courtesy will positively influence PSSB*.

### 2.4. Mediating Role of OBSE

The present study adds to the knowledge base on customer courtesy by investigating the link between customer courtesy service and PSSB of service employees, focusing particularly on the mediating role of OBSE. Self-consistency theory [24] has typically been used to examine how individuals’ self-concepts influence their attitudes and behaviors at work (e.g., [71]). Drawing on self-consistency theory, employees with high OBSE are motivated to maintain a positive self-image. This theory posits that individuals strive for consistency between their self-perceptions and their actions. Consequently, employees with a high OBSE are likely to demonstrate effective job performance in order to uphold their self-belief as valuable contributors to the organization. By considering customer courtesy as a job-related task accomplishment and employing Korman’s self-consistency theory, it can be concluded that customer courtesy increases PSSB by enhancing an individual’s sense of self-worth in the organization [24]. According to the self-consistency theory, high-OBSE employees are more inclined to adopt attitudes and act in ways that support their high OBSE. Pierce and Gardner reported that employees with high OBSE display higher levels of extra-role performance [22]. Accordingly, we predict that customer courtesy positively influences PSSB through increased OBSE. Also from the previous sections, customer courtesy positively influences OBSE, and OBSE positively influences PSSB. Hence, we propose:

**H4.**  *OBSE mediates the customer courtesy–PSSB link*.

### 2.5. FAW as a Moderator

The literature has focused on employees’ thoughts while at work, what precedes them, how it affects them, and how they react to organizational experiences [72]. According to Lorence and Mortimer, when employees psychologically identify with their work, they are likely to spend a lot of time reflecting on their (pleasant) work rather than on things that are perceived as less enjoyable or less psychologically involved [73]. The construct “focus of attention at work” was put forward by Gardner et al., and they explained how it was linked to other organizational-related variables [17]. They asserted that an employee’s reaction to an organizational condition is a function of how much attention he or she devotes to it. FAW is an individual difference construct that indicates employees’ FAW differs greatly depending on which categories of phenomena they think about and how much they think about them. Gardner et al. defined “focus of attention” as “*an employee’s cognitive orientation towards each of many targets while he or she is at work* [17].” It describes the amount of mental capacity (or effort) devoted to a certain goal or phenomenon [74]. Individual job focus can be driven by both positive and negative factors. On the positive side, Ho et al. and Pollack et al. identified that employees with high work “passion” or “enthusiasm” tend to exhibit a heightened FAW compared to their counterparts [75,76]. Conversely, negative experiences such as role conflict can also lead employees to devote greater attention to their job duties.

Gardner et al.’s and Siegall and McDonald’s empirical studies demonstrated that the FAW impacts how employees perceive their work environment as well as how they respond to those impressions after they have been established [17,77]. Gardner et al. found that FAW moderated the relationship between work environment factors and job satisfaction [17]. Building on prior research, Siegall and McDonald found a positive correlation between employee job focus and work engagement [77]. This implies that the level of attention employees dedicate to their work tasks might function as a moderating variable, influencing the relationship between workplace environment characteristics and employee work outcomes. In other words, a work-related stimulus that gets employees’ attention will not be effective if they are not thinking about it. Employees exhibiting a high FAW are likely to be more receptive to their working environment’s conditions (e.g., customer courtesy) that foster a higher level of OBSE. Based on Swann et al.’s self-verification theory [78], we argue that customers’ courtesy will be accompanied by an increasing level of OBSE in individuals with a high level of FAW, consistent with belief in their organizational worth. Additionally, employees with high job focus have better cognitive clarity and are more cognizant of their working environment. Based on these discussions, it can be contended that employees’ FAW will act as a potential boundary condition influencing the link between customer courtesy and OBSE. Therefore, we believe that employees with a high FAW are more perceptive and will have a deeper comprehension of the messages conveyed to them from their workplace regarding their organizational worth, which will affect their OBSE.

Additionally, and as discussed earlier, the literature has documented that OBSE has a positive association with constructive work behavior and attitude [79]. It is proposed that FAW can strengthen or weaken this relationship. This study posits a link between employee job focus and self-verification processes. We expect that employees who exhibit a high degree of FAW, dedicating a significant portion of their time to contemplating work-related matters, will demonstrate a stronger tendency to engage in self-verification using work-environment-derived attitudes as reference points. In simpler terms, employees who are highly focused on work are more likely to seek confirmation of their self-perceptions by referencing the attitudes and expectations present within their work environment. In a way, the workplace serves as the setting for self-verification, and its effects are amplified when employees are preoccupied with it most of the time. On the other hand, employees with a low FAW are less likely to self-verify their work-related attitudes and behaviors. Therefore, we contend that employees who are preoccupied with work-related matters are more susceptible to aligning their work attitudes and behaviors with their level of OBSE. They will display a stronger link between OBSE and work-related outcomes than employees with a low FAW. We propose that employee FAW functions as a potential moderator in the relationship between OBSE and work-related attitudes and behavioral intentions. For employees exhibiting high work focus, their existing OBSE is more likely to translate into job-related attitudes (encompassing “cognitions”, “emotions”, and “behavioral intentions”) that reinforce and elevate their self-perceptions. In simpler terms, employees who are highly attentive to their work are more susceptible to developing work-related mindsets and behaviors that confirm and strengthen their existing sense of self-worth within the organization. Thus, we hypothesize the following:

**H5a.**  *FAW moderates the relationship between customer courtesy and OBSE, such that the relationship is positive and stronger for employees with high FAW*.

**H5b.**  *The relationship between OBSE and PSSB is stronger for employees with a high FAW than for employees with a low FAW*.

**Figure 1:**
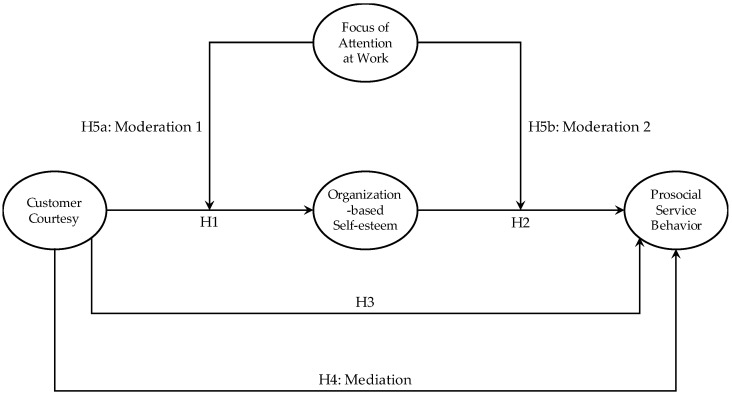
Research framework.

The proposed relationships are shown in Figure 1.

## 3. Methods

### 3.1. Participants and Data Collection Procedure

Participants in this study were full-time customer-contact employees from the South Korean hospitality industry. The researchers contacted the top management or human resources managers of the hotels to explain the objectives of the study, which aimed to investigate the impact of customer courtesy on employees’ prosocial service behavior, with a focus on the mediating role of organization-based self-esteem and the moderating role of the focus of attention at work. Additionally, the survey protocol was thoroughly explained, ensuring that all ethical guidelines and confidentiality measures were clearly communicated. Out of those contacted, nine upscale hotels in major cities across South Korea, including Seoul, Busan, and Jeju, volunteered to participate in this research endeavor. These hotels represent a mix of luxury and business categories, with varying sizes ranging from approximately 150 to over 500 rooms. This diverse selection provides a comprehensive representation of the South Korean hotel industry. After developing the survey, it was initially pretested with a small number of hotel employees to ensure the participants understood the questions. A paper-based survey was used for data collection. Each survey had a cover letter explaining the purpose of the research and the contact details of the principal investigator. To ensure participant confidentiality and anonymity, all responses were treated with strict discretion. Hotel human resource managers facilitated the survey distribution. The human resource managers were requested to distribute the surveys to a wide group of customer-contact employees in their hotel firms to reduce the risk of selection bias. The participants received the survey in a sealed envelope. Upon completion, participants were instructed to seal the completed surveys within the provided return envelopes and deposit them in designated locked drop boxes positioned near the reception areas of their respective hotel workplaces. Out of the 600 surveys distributed, we received 423 filled-in responses, out of which 401 were usable for further analysis. The sample consisted of 401 participants, with a gender distribution of 58% male and 42% female. The average participant age was 33.2 years. In terms of educational attainment, the majority (41%) held a bachelor’s degree, followed by those with an associate degree (36%). A smaller proportion had a high school diploma (17%), and a few (6%) held professional qualifications, including master’s and doctorate degrees. In terms of departmental distribution, housekeeping staff comprised 19% of the respondents, followed by front office (28%), food and beverage (29%), and recreation (24%). The average employee tenure with the current hotel firm was 5.6 years, with a maximum of 8 years. The correlations between the investigated constructs were in the anticipated direction.

### 3.2. Measures

All the constructs were measured using scales that were adapted from previous literature. All the constructs included in this study are measured using reflective measurement logic.

Customer courtesy: It was assessed using five items from Yoon et al.’s study [15]. A sample item includes the following: “*My customers usually act courteously to me.*” The respondents responded to a seven-point Likert-type scale ranging from “1-strongly disagree” to “7-strongly agree”.

OBSE: Ten items from Pierce et al.’s OBSE scale were employed to assess employees’ level of organization-based self-esteem [16]. The items were rated on a 5-point Likert-type scale ranging from “1-strongly disagree” to “5-strongly agree”. A sample item includes the following: “*I can make a difference around here*”.

PSSB: Five items specifically from the “*extra-role customer service*” dimension of Bettencourt and Brown’s study were used to measure PSSB [6]. These items were chosen because they most directly reflect the discretionary, above-and-beyond behaviors that are central to our research focus [15]. A sample item includes the following: “*Often goes above and beyond the call of duty when serving customers*”. The items were anchored on a seven-point Likert-type scale ranging from “1-strongly disagree” to “7-strongly agree”.

FAW: Gardner et al. developed the “focus of attention at work” scale [17]. It was designed to reduce common method bias by employing a non-traditional item format. Employees were requested to specify how much they “think about” each of the following focus targets (“job”, “work unit”, and “off-job”) while they are at work (e.g., “how often you think about job factors while at work”). The employees were required to indicate their responses on 13 cm lines anchored from “almost never” to “almost all the time”.

### 3.3. Common Method Bias

Due to the cross-sectional design nature of the study, both procedural and statistical remedies were employed to reduce common method bias [80]. As procedural remedies, the participants were assured of anonymity and that there were no right or wrong answers. Additionally, high-quality measures were adopted, ambiguous terms were avoided, and the items across constructs were mixed. Furthermore, as a statistical remedy, we employed the marker technique suggested by Lindell and Whitney to determine potential common method bias issues [81]. Additionally, the full collinearity test demonstrated that the variance inflation factor values were less than 3.3 [82]. The results suggested that common method bias was unlikely to have a substantial influence on the study’s outcomes.

### 3.4. Control Variables

As suggested in the previous study [59], gender, educational level, and tenure were controlled in this study. Although the study operationalizes the constructs as individual-level variables, the data structure is nested. Therefore, we conducted a one-way ANOVA to test the non-independence of errors by treating hotel as the independent variable and customer courtesy, OBSE, and PSSB as the dependent variables.

## 4. Results

### 4.1. Measurement Model Results

The approach suggested by Anderson and Gerbing was adopted [83], and SmartPLS 4 was used to analyze the data [84]. Since the items are reflective, they were assessed for their reliability and validity [85]. Utilizing Cronbach’s alpha and composite reliability, internal consistency was assessed. Item loading was used to evaluate the indicator’s reliability, and the value of the average variance extracted (AVE) was used to examine the indicator’s convergent validity. The loading of a few items was below the recommended threshold of 0.7. However, since the AVEs were above 0.5, all the items were retained [85]. The reliability values were well above the recommended cut-off of 0.7. All of the constructs’ AVE values, which measure convergent validity, were greater than the suggested threshold of 0.5. Table 1 shows the validity and reliability results.

To assess discriminant validity, Fornell-Larker’s criterion was used [86]. The square root of the AVEs was compared with the latent variable correlations and has to be greater than them. As indicated in Table 2, the square roots of AVEs are greater than the correlations with the other constructs, showing discriminant validity was achieved.

### 4.2. Structural Model Results

**Figure 2:**
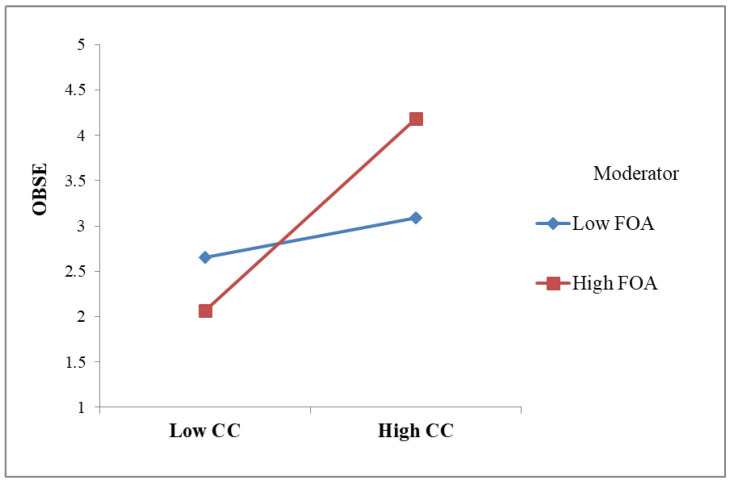
Moderating effect of FAW on the relationship between customer courtesy and OBSE.

**Figure 3:**
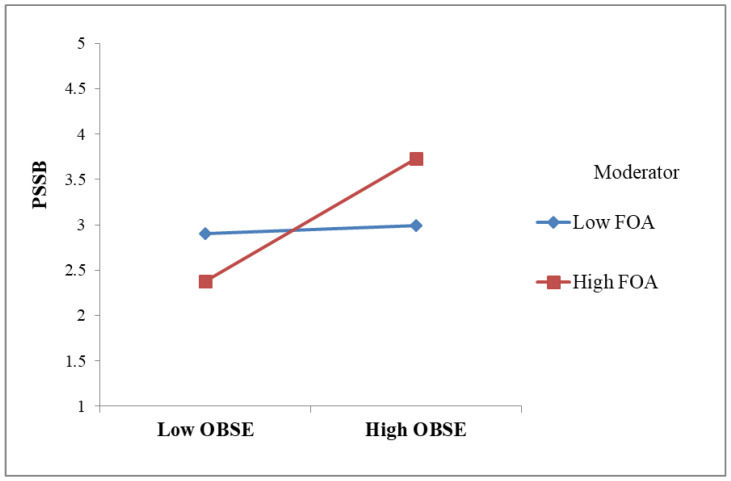
Moderating effect of FAW on the relationship between OBSE and PSSB.

To test the hypotheses, the β values and associated t-values were examined by utilizing a bootstrapping approach with a resample of 5000 [85]. H1 proposed that customer courtesy had a positive effect on OBSE. As expected, the results indicated that customer courtesy was a significant predictor of OBSE (β = 0.637, *p* < 0.001, R^2^ = 0.406), supporting H1. OBSE had a significant positive influence on PSSB (β = 0.359, *p* < 0.001, R^2^ = 0.513), supporting H2. H3 suggested customer courtesy is positively related to PSSB. This was supported (β = 0.432, *p* < 0.001). To investigate the mediating role of OBSE, we examined the 95% bias-corrected confidence interval [87]. The estimated indirect effect of customer courtesy on PSSB through OBSE is significant (0.227, 95%CI [0.168, 0.293]). Thus, H4 was supported. In hypothesis H5a, we proposed that FAW moderated the link between customer courtesy and OBSE. The moderating effect was significant (β = 0.420, [0.413, 0.447], ∆R^2^ = 0.13, *p* < 0.001), supporting H5a. To interpret the moderation pattern, simple slopes were plotted, as shown in Figure 2. From Figure 2, it is understood that FAW strengthened the positive link between customer courtesy and OBSE. Similarly, H5b hypothesized that FAW moderated the OBSE–PSSB link. The moderating effect was significant (β = 0.316, [0.192, 0.821], ∆R^2^ = 0.16, *p* < 0.001), supporting H5b. From the simple slopes in Figure 3, FAW strengthened the positive relationship between OBSE and employees’ PSSB. The results are summarized in Table 3.

## 5. Discussion and Implications

### 5.1. Discussion

Interacting with customers is an integral part of the job of the front-line hotel staff. This separates the hotel and its operational processes from non-service-intensive organizations. The present study explored the mediating mechanism of OBSE and the boundary condition of FAW in the association between customer courtesy and PSSB. Consistent with Yoon et al.’s finding, customer courtesy promoted PSSB among customer-contact employees [15]. The results demonstrated that positive experiences with customers can serve as a reinforcer. Hotel firms do not view customers as passive recipients of services but rather as co-creators of the service. A customer’s attitudes and moods toward a service provider can affect the success or failure of the service experience [88]. By investigating the social interaction between customers and customer-contact employees, the study addresses an important theoretical gap that has significant practical implications for the hospitality industry. The results demonstrate that OBSE mediates the link between customer courtesy and PSSB. The findings align with previous studies demonstrating the importance of self-evaluation at work (i.e., OBSE) as a significant source of intrinsic motivation leading to improved employee behavioral outcomes [16,59]. Thus, this study expounds on “why” customer courtesy contributes to employees’ PSSB. Employee self-evaluations serve as a critical determinant of their work behaviors [89]. Thus, when employees gain a sense of self-worth at work, customer courtesy can promote their PSSB. Employees’ FAW moderated the customer courtesy–OBSE link and between OBSE and PSSB. The results align with Gardner and Pierce’s assertion that job focus strengthens the influence of job characteristics on OBSE development [56]. This study further extends these findings by demonstrating that high FAW intensified the positive effect of OBSE on PSSB.

### 5.2. Theoretical Contributions

The study contributes to the hospitality literature by empirically exploring customer courtesy, which was introduced as a construct in the prior study [15]. The study unraveled the underlying process and the contextual factors that moderate the link between customer courtesy and employees’ PSSB. Several studies in the past have documented that customers are a source of incivility in the hospitality industry [12,90,91] and demonstrated how they diminish service performance and employees’ psychological resources. Similarly, the prior scholars investigated how discretionary customer behavior affected customers [92,93]. However, the findings showed that customers can be a source of encouragement and support. The findings revealed that in the South Korean hospitality sector, customers’ positive responses are crucial in boosting customer-contact employees’ PSSB. Therefore, by investigating the ways in which customer support and relationship-building behaviors influence customer–employee interactions, particularly in pseudo-relationships, we further contribute to the existing body of knowledge on service management [18,94]. To the best of our knowledge, this study is among the first to suggest that customers have a significant influence on customer-contact employees’ perceptions of and behaviors during service interactions. Most service literature is based on the service-profit chain and focused on how customer contact employees’ attitudes and behaviors may affect customers’ service experiences [95,96]. But according to our research, customers, especially those who are courteous, can contribute to positive customer–employee interactions, which account for customer-contact employees’ positive emotions. In stressful service jobs, this may be a great opportunity to re-energize and gain psychological resources. Given the principle of reciprocity inherent in social interactions, customer-facing employees are likely to reciprocate prosocial customer behavior with their own PSSB. The fact that customers can boost customer-contact employees’ PSSB promises a prospective future inquiry regarding how hotel firms may use external resources (server-friendly and courteous customers) to promote PSSB.

Second, the types of self-esteem that are pertinent to the work environment when service employees interact with customers have not been taken into account by the prior researchers [97]. Self-esteem has been viewed as a multi-layered construct with varying degrees of specificity [22,98]. In other words, people differ in their degrees of competence and self-worth in a range of social circumstances and positions [24]. Thus, it is conceptually crucial to consider the environment in which self-esteem is built since, depending on the amount of specificity, self-esteem may have varied effects on people’s attitudes and behaviors. OBSE reflects a context-dependent perception of self-worth that demonstrates heightened susceptibility to influence from organizational factors and job-related experiences [22]. Hence, it could serve as a mediator in the customer courtesy–employees’ PSSB link. Additionally, customer courtesy promotes psychological states in employees that contribute to PSSB. Specifically, when customer-facing employees experience courteous interactions, it can enhance their OBSE. This heightened OBSE, in turn, motivates them to engage in PSSB. This pattern reflects Barnes et al.’s and Xu et al.’s assertion that the interactional aspect of service experience determines employees’ behavior [99,100]. Given that the underlying mechanism in the association between customer courtesy and PSSB is under-researched, understanding the role of OBSE as a mediator is important. Therefore, delineating why OBSE mediates the link between customer courtesy and PSSB has theoretical significance.

Third, this study sheds significant light on customer-contact employees’ PSSB. An essential construct in positive organizational scholarship for the service sectors is PSSB [6]. Previous studies have focused on negative factors influencing employees’ prosocial behavior [12]. In the hospitality industry, exploring the factors that drive PSSB has considerable significance [10]. Accordingly, the previous studies by Hsu and Lai (2024) and Jin et al. (2021) have called for further research to identify variables that boost customer-contact employees’ PSSB [101,102]. The present study addressed this by examining customer courtesy as an antecedent of PSSB. Next, this is one of a select few studies that established a relationship between employee FAW and OBSE. The findings align with the person-situation interaction perspective, which highlights the combined effect of individual employee characteristics and situational variables on work performance [15]. The findings further validate the moderating role of FAW in a non-Western/developing country context.

### 5.3. Practical Implications

The study offers several managerial implications for the hospitality industry. First, the findings demonstrated that customer courtesy positively influences OBSE and PSSB. Therefore, the managers may want to explain to the customer-contact employees and clients how they specifically contribute to enhancing service delivery [103]. Second, due to its close relation to organizational phenomena and its potential to explain organizationally relevant behaviors better than other constructs of self-evaluation, including global self-esteem and generalized self-efficacy, the study focused on OBSE. Employee OBSE should be a priority for organizations seeking to increase engagement in PSSB. High-OBSE employees believe they play an important and competent role within their organization [104]. Employees who have this kind of positive self-concept are more likely to be inspired to act in ways that benefit the firm. In the workplace, self-evaluations and self-perceived competencies may be socially influenced and molded by interactions with others [59]. In other words, employees’ views of their value as organizational members may be shaped by how consumers treat them [15]. Furthermore, supervisors may reinforce the importance of customer-contact employees to bolster their self-esteem and, consequently, their OBSE. Supervisors must take into account and, if feasible, improve the elements that support OBSE. This entails taking steps to ensure that employees have meaningful success at work, including empowering them, giving them feedback on their performance, and coaching them. Employees will spend more of their attention (high job focus) on their responsibilities if they feel their roles in their companies are important and enjoyable, which will lead to high OBSE [75]. Third, this study has significant ramifications for hiring and training service employees. The results demonstrate that when selecting employees, employees with a high FAW can be selected. A heightened FAW may be achieved through job redesign that produces more stimulating employment. We suggest that supervisors consciously bring up favorable role attributes in conversations with employees to verbally reinforce them. Casual comments on the autonomy and resources that the employees have at their disposal to help them succeed at work may encourage employees to focus their attention at work [72,105]. Supervisors may also highlight knowledge and abilities that are pertinent to employees’ performance at work. Such interactions will also amplify the development of OBSE. Fourth, the results demonstrated that customer courtesy improved customer-contact employees’ PSSB. Hotels can, thus, think of creative methods to reward customers for their courteous behavior by introducing both financial (e.g., discounts) and non-financial incentives. Hotels could also inform customers about the advantages of such collaboration through their websites and social media platforms. For instance, hotels may develop video clips combined with textual and graphical resources to show customers how their courteous behavior can promote PSSB. Additionally, to encourage customer courtesy, hotels might use persuasive methods (such as Cialdini’s principles of persuasion) while designing these resources [61].

### 5.4. Limitations and Future Research Directions

Despite its contributions, the study has its shortcomings. First, a cross-sectional survey design was employed, and the data came from a single source, suggesting they are susceptible to common method bias [80]. This prevents making definite claims regarding the causal inference. Although method bias was not a concern in the present study, future studies may utilize experimental designs and observer ratings of PSSB to aid in determining the causal effects of customer courtesy on PSSB. Second, the study is specific to the South Korean hospitality industry context, and South Korea is a collectivistic society that values moral behavior [13]. The behavioral responses of customer-contact employees may be influenced by this cultural context [106]. Therefore, the proposed relationships may be validated in other cultural settings, and caution should be exercised when attempting to generalize the findings. Third, empirical studies on customer courtesy are still in the infancy stage. Further studies are required to understand the motivational and behavioral responses to customer courtesy. Customer-contact employees who engage in high-level customer–employee interactions feel emotionally uplifted in addition to experiencing positive social exchange with their clients [107]. According to affective events theory, emotional experiences at work predominantly take the form of emotional responses to incidents at work, which, in turn, influence employees’ motivation and performances [108]. Therefore, customer courtesy may influence employees’ positive affect and intrinsic motivation. Future studies may examine these relationships. Finally, we focused on the positive impact of customer courtesy on PSSB. However, this approach limits our understanding of how negative customer behaviors, such as customer insults and bad behavior, might affect PSSB. These negative interactions are likely to have significant implications for employee well-being and performance. Therefore, future research could address this limitation by investigating the effects of such negative customer behaviors on PSSB. This line of inquiry would not only complement our findings but also provide a more holistic view of the dynamics between customer behavior and employee responses in the service industry.

The authors should discuss the results and how they can be interpreted from the perspective of previous studies and the working hypotheses. The findings and their implications should be discussed in the broadest context possible. Future research directions may also be highlighted.

## Figures and Tables

**Table 1:** Item loadings and reliability.

Constructs and Items	Loadings	Cronbach’s α	Composite Reliability	AVE
Customer courtesy		0.816	0.872	0.578
“My customers usually act courteously to me.”	0.667			
“My customers are cooperative with me so that I can serve them easily.”	0.755			
“My customers are well-mannered.”	0.798			
“My customers usually treat me with warmth.”	0.835			
“My customers like me as a service employee.”	0.734			
Organization-based self-esteem		0.933	0.944	0.629
“I count around here.”	0.845			
“I am taken seriously around here.”	0.853			
“I am important around here.”	0.855			
“I am trusted around here.”	0.793			
“There is faith in me around here.”	0.814			
“I can make a difference around here.”	0.877			
“I am valuable around here.”	0.810			
“I am helpful around here.”	0.758			
“I am efficient around here.”	0.646			
“I am cooperative around here.”	0.642			
Prosocial service behavior		0.913	0.936	0.744
“Voluntarily assists customers even if it means going beyond job requirements.”	0.750			
“Helps customers with problems beyond what is expected or required.”	0.905			
“Often goes above and beyond the call of duty when serving customers.”	0.886			
“Willingly goes out of his/her way to make a customer satisfied.”	0.898			
“Frequently goes out the way to help a customer.”	0.865			
Focus of attention at work		0.801	0.879	0.709
“How often you think about job factors while at work.”	0.869			
“How often you think about work unit factors while at work.”	0.893			
“How often you think about off-the-job factors while at work.”	0.761			

**Table 2:** Discriminant validity.

	CC	OBSE	PSSB	FOA
Customer courtesy	**0.760**			
Organization-based self-esteem	0.636	**0.793**		
Prosocial service behavior	0.661	0.635	**0.863**	
Focus of attention at work	0.173	0.353	0.297	**0.842**

Note: Values in bold are the square root of AVEs. Below the diagonals are the correlation values.

**Table 3:** Summary of hypotheses testing.

Paths	Path Coefficient	t-Value	Result
H1: Customer courtesy → OBSE	0.637	20.137 **	Supported
H2: OBSE → PSSB	0.359	8.117 **	Supported
H3: Customer courtesy → PSSB	0.432	10.746 **	Supported
H4: Customer courtesy → OBSE → PSSB	0.227	CI [0.168, 0.293]	Supported
H5a: Customer courtesy × FAW → OBSE	0.420	5.092 **	Supported
H5b: OBSE × FAW → PSSB	0.316	3.359 **	Supported

Note: ** *p* < 0.001.

## Data Availability

The raw data supporting the conclusions of this article will be made available by the authors on request.
